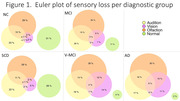# Alterations in brain structure and functional connectivity associated with hearing loss and olfactory loss

**DOI:** 10.1002/alz.086734

**Published:** 2025-01-03

**Authors:** Natalie Phillips, Paul T. Best, Nicole Grant, Ameera Kabir

**Affiliations:** ^1^ Concordia University, Montréal, QC Canada; ^2^ Concordia University, Montreal, QC Canada

## Abstract

**Background:**

Sensory loss in hearing, vision, and olfaction are highly prevalent in older adults and are each associated with a higher risk of developing dementia. This study sought to identify the extent to which these sensory factors are associated with alterations in brain function and structure older adults with or at risk for dementia. We examined groups who range from relatively low risk (those with normal cognition and no cognitive complaints (NC)), to those with higher risk, namely individuals with subjective reports of cognitive decline (SCD) but normal cognition and those with mild cognitive impairment (MCI).

**Method:**

We used data from the Comprehensive Assessment of Neurodegeneration and Dementia (COMPASS‐ND) study (Release 7). Hearing loss was assessed with a pure‐tone screening protocol, vision was assessed with the MARS test for contrast sensitivity, and olfaction was assessed with the Brief Smell Identification Test (BSIT). We examined the frequency of sensory deficits in 128 NC (mean age = 69 years; mean education = 16 years), 135 participants with SCD (age = 70; education = 17), 241 with MCI (age = 72; education = 16), and 93 with Alzheimer’s disease (AD, age = 75; education = 15). Participants were matched on age and education, except for AD participants who were older.

**Results:**

As shown in Figure 1, normal sensory performance in all three modalities was observed in the minority of participants and decreased in prevalence across the dementia risk spectrum groups (e.g., NC: 29%; SCD: 29%; MCI: 11%; AD: 2%). Olfactory deficits (hyposmia or anosmia) were the most frequent (ranging from 45% in NC to 61% in AD), followed by hearing impairment, and then deficits in visual contrast sensitivity. Deficits in multiple sensory domains were highly prevalent, with 37% of participants with AD having deficits in two or more domains. Preliminary analyses indicate that hearing loss is associated with altered connectivity in the default mode network in MCI participants and olfactory loss is associated with reduced hippocampal volumes in SCD.

**Conclusion:**

These analyses indicate that sensory loss is highly frequent and co‐morbid in persons with or at risk for dementia. These findings have implications for cognition, brain function, functional activities, and care delivery for persons with or at risk for dementia.